# How views of oncologists and haematologists impacts palliative care referral: a systematic review

**DOI:** 10.1186/s12904-020-00671-5

**Published:** 2020-11-23

**Authors:** Naveen Salins, Arunangshu Ghoshal, Sean Hughes, Nancy Preston

**Affiliations:** 1 Department of Palliative Medicine and Supportive Care, Kasturba Medical College, Manipal Academy of Higher Education, Manipal, Karnataka 576104 India; 2 Department of Palliative Medicine, Tata Memorial Centre, MB-G-75, DR E Borges Road, Parel, Mumbai, 400012 India; 3 Division of Health Research, Faculty of Health and Medicine, Furness College, Lancaster University, C051, C – Floor, Bailrigg, LA1 4YW UK

**Keywords:** Haematologists, Oncologists, Palliative care, Referral, Views

## Abstract

**Background:**

Worldwide, many patients with cancer, are infrequently referred to palliative care or are referred late. Oncologists and haematologists may act as gatekeepers, and their views may facilitate or hinder referrals to palliative care. This review aimed to identify, explore and synthesise their views on referrals systematically.

**Methods:**

Databases of MEDLINE, CINAHL, PsycINFO, EMBASE, Scopus, Web of Science and Cochrane were searched for articles from 01/01/1990 to 31/12/2019. All studies were scored for their methodological rigour using Hawker’s tool. Findings were synthesised using Popay’s narrative synthesis method and interpreted using a critical realist lens and social exchange theory.

**Results:**

Out of 9336 initial database citations, 23 studies were included for synthesis. Five themes were developed during synthesis.

1. *Presuppositions of oncologists and haematologists about palliative care referral:* Role conflict, abandonment, rupture of therapeutic alliance and loss of hope were some of the presuppositions that hindered palliative care referral. Negative emotions and perception of self-efficacy to manage palliative care need also hindered referral.

2. *Power relationships and trust issues:* Oncologists and haematologists preferred to gatekeep the referral process and wished to control and coordinate the care process. They had diminished trust in the competency of palliative care providers.

3. *Making a palliative care referral: A daunting task:* The stigma associated with palliative care, navigating illness and treatment associated factors, addressing patient and family attitudes, and overcoming organisational challenges made referral a daunting task. Lack of referral criteria and limited palliative care resources made the referral process challenging.

4. *Cost-benefit of palliative care referral:* Pain and symptom management and psychosocial support were the perceived benefits, whereas inconsistencies in communication and curtailment of care were some of the costs associated with palliative care referral.

5. *Strategies to facilitate palliative care referral:* Developing an integrated model of care, renaming and augmenting palliative care resources were some of the strategies that could facilitate a referral.

**Conclusion:**

Presuppositions, power relationships, trust issues and the challenges associated with the task of referrals hindered palliative care referral. Oncologists and haematologists appraised the cost-benefit of making a palliative care referral. They felt that an integrated model of care, changing the name of palliative care and augmenting palliative care resources might facilitate a referral.

**Supplementary information:**

**Supplementary information** accompanies this paper at 10.1186/s12904-020-00671-5.

## Background

Worldwide, the majority of patients with cancer present in late stages of illness and need palliative care [1]. However, they are infrequently referred to palliative care or are referred late [2–5]. Early palliative care referral is associated with improved quality of life, symptom control, treatment decision making, advance care planning, end of life care and reduced costs [6–8]. In a cancer care setting, oncologists and haematologists may act as gatekeepers, and their views about palliative care referral may facilitate or hinder referral to palliative care [9, 10]. This research aimed to explore the views of oncologists and haematologists on specialist palliative care referral.

A scoping review of the multidisciplinary database Scopus [11], identified previous systematic reviews on palliative care referral. These reviews looked at barriers to accessing palliative care [12], referral patterns [13], referral criteria [14], and appropriateness of referral [15]. Other related systematic reviews looked at interventions to improve palliative care referral [16], integration of oncology and palliative care [17], the effect of age of the patient on palliative care referral [18], and collaboration between generalist and specialist palliative care teams [19]. Although there are studies about the views of oncologists and haematologists on palliative care referral, these studies have not been reviewed systematically necessitating this systematic review.

## Methods

This research aimed to systematically identify, explore and synthesise the views of oncologists and haematologists on specialist palliative care referral. We conducted this systematic review with a review question as “What are the views of oncologists and haematologists on palliative care referral”? The review question was formulated using **P**opulation, Phenomenon of **I**nterest and **Co**ntext (PICo) framework [20]. The population studied were oncologists and haematologists; the phenomenon of interest was views on specialist palliative care referral, and the context was the cancer care setting.

The results of the scoping search showed that the typology of evidence informing this review is a heterogeneous mixture of surveys, qualitative studies, and mixed-method studies. Popay’s narrative synthesis method was chosen as the review approach as it is appropriate for synthesising textual data from surveys and qualitative studies into themes [21]. Moreover, it facilitates using a theoretical framework for interpreting study findings [21]. The literature was reviewed using a critical realist lens [22], and the findings were interpreted using social exchange theory [23]. Critical realist approach involves documenting the empirically known phenomenon of referral and going beyond the empiric observations to explain the actual events and generative mechanisms [24]. Social exchange theory is the theorisation of the social behaviour of exchange where people are motivated to engage in an exchange where they may gain or forfeit something of value [23].

The systematic review protocol was registered with the Centre for Reviews and Dissemination, University of York. The PROSPERO registration number was CRD42018091481.

### Search strategy

The review question was divided into search concepts, and a scoping search helped to identify the key search terms relevant to each concept of the review question. The scoping search also helped to identify three index papers to test the sensitivity of the search [25–27]. The index papers facilitated the expansion of the search terms to find free-text and thesaurus terms related to the scope of the review [28]. Four subject-specific databases (MEDLINE, CINAHL, PsycINFO and EMBASE) were searched by combining free-text and the thesaurus terms specific to the database using the Boolean operators [29]. Three multidisciplinary databases (Scopus, Web of Science and Cochrane database) were searched using free-text terms. Additional file 1 provides information about the thesaurus and free text terms used in this review.

Studies published in English involving human subjects from 01/01/1990 to 31/12/2019 was accessed. The article search was limited from 1990 onwards as the first published literature on palliative care in the MEDLINE dated back to 1993 [30]. Additional file 2 provides the list of eleven journals hand searched for additional citations. They were chosen based on the scoping review. The bibliographies of the full-text articles included in the review were checked to make sure that no relevant studies were missing [31]. The citations of the included publications were searched using Google Scholar and Web of Science to identify more articles pertaining to the review. The articles identified through citation searching were checked for their citations until the search led to no additional relevant articles [32].

### Study eligibility and scoring for methodological rigour

The selection criteria of the studies included in the review are listed in Table 1. All studies were scored for their methodological rigour using Hawker’s tool [33]. Additional file 3 provides information about Hawker’s tool [33]. Hawker’s tool allows methodological scoring of a mixed typology of studies and a growing number of palliative care systematic reviews have used this tool [12, 19, 34]. Scoring is based on the nine criteria set by the Hawker provided in Additional file 3. Each criteria is assigned a score between 1 to 4 (1 = very poor and 4 = good), and 9 is the minimum score, and 36 is the maximum score [33]. Only those studies scoring 19 and above were included in the review. Although it has not provided a cut-off score for inclusion, previous palliative care systematic reviews have used a score of 19 for inclusion [19, 35]. Three studies were excluded from the review as they scored less than 19 in Hawker’s score [33]. The minimum score of the studies included in this review was 25, and the average score was 30.

**Table 1:** Systematic Review Inclusion and Exclusion Criteria

Inclusion Criteria	Exclusion Criteria
1. Empiric research on human subjects published in the English in a peer-reviewed journal after 1990. 2. Studies exploring views of the oncologists, haematologists and cancer specialists about palliative care referral. 3. Studies with Hawker’s methodological quality score of 19 or above.	1. Empiric research on the effectiveness of palliative care referral or mechanisms underpinning the effectiveness of the referral. 2. Studies conducted at a non-cancer setting and not involving oncologists and haematologists

### Data extraction

The screening, quality appraisal and data extraction were conducted independently by two reviewers. The data extraction sheet provided in Additional file 4 has five sections. The initial section had information regarding the country and year of publication. The second section focused on the type of study, that is a survey, qualitative or mixed-method. In this section, study objectives, population and study setting were also described. Study sample, participants, inclusion and exclusion criteria, research design and methods were elucidated in the third section. The fourth section provided information on study findings and conclusions. The last section discussed the strengths and limitations of the study and biases.

### Data synthesis

During Popay’s narrative synthesis, the first step was to identify a theoretical framework for the review that can contribute to the interpretation of review findings [21]. The theoretical lens of social exchange theory was used to interpret the themes generated during the synthesis [23]. The second step was to develop a preliminary synthesis. A preliminary synthesis was generated by providing a brief textual description of the studies informing the review. The studies informing this review were grouped according to the country, type of population studied and the factors influencing referral. The textual description helped the reviewers to be familiar with the data before analysis. The third step was to explore relationships within and between studies. The relationships were explored by representing the study findings as meaningful categories and themes and creating a thematic map. During this step, the reviewers also explored the heterogeneity of the included studies in terms of population, setting and typology. The fourth step was to assess the robustness of synthesis. It was done by critically reflecting the synthesis process and providing information about the limitation of the synthesis and possible sources of biases [21].

## Results

### Overview of studies

**Fig. 1:**
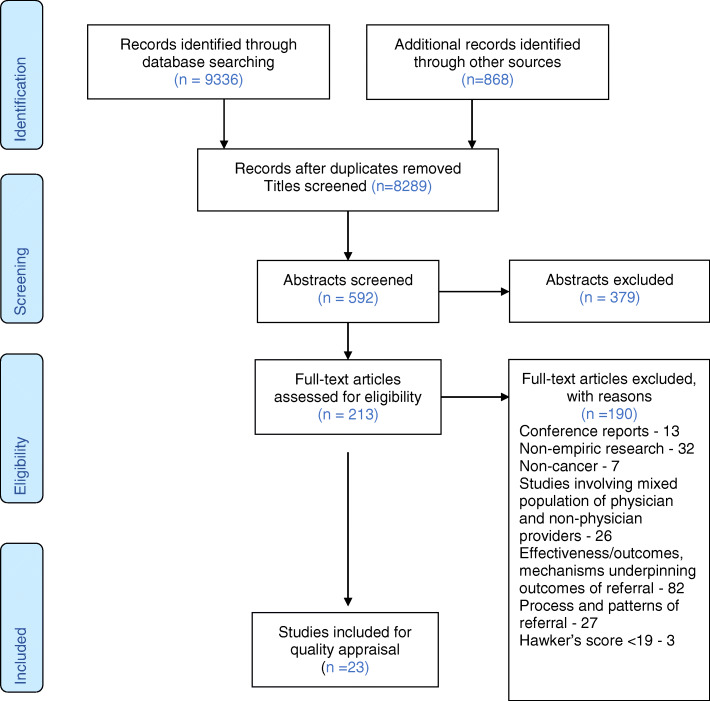
PRISMA flow diagram

Out of 9336 initial database citations, 23 studies were included for synthesis. The PRISMA flow diagram for this review is provided in Fig. 1. Ten studies were qualitative, ten were surveys, and three were mixed-method studies. Twelve studies were from North America (nine USA and three Canada), seven from Europe (three from France, one each from Belgium, United Kingdom, Hungary and Cyprus), two from Australia and two from Asia (one each from Japan and Israel). The majority of the surveys and mixed-method studies were multi-centre studies spanning across several centres within the country and across countries. Moreover, they were often linked to professional cancer societies. The qualitative studies were mostly limited to one or more centres within a region. A detailed overview of the studies can be found in Table 2. There were only two studies on paediatric referrals [49, 53]. One had a mixed adult and paediatric population [49] and another a qualitative study of paediatric oncologists [53].

**Table 2:** Overview of the Studies included in the Systematic Review

Author (Year) Country	Research question	Participants/Setting	Method	Key findings	Hawker Score
Horlait et al. (2016) [36] Belgium	What are the barriers to introduce palliative care into discussion with patients with advanced cancer?	15 medical oncologists from academic and non-academic hospitals	Grounded theory	Following were the barriers identified by the oncologists. Physician related barriers were emotional bonding with the patients, feeling of professional failure, discomfort with death and dying and lack of experience and training in managing patients with advanced illness. Patient related barriers were language and culture, denial, and unrealistic expectations. Family related barriers were protection of patients, and family disputes. Disease related barriers were lack of clear guidelines for palliative care referral, unpredictable trajectory of illness and unexpected progression. Organisation related barriers were lack of availability of palliative care services, excessive focus on cure, lack of space and time to discuss palliative care, and excessive workload. The societal barrier was the stigma associated with palliative care and palliative care referral.	34
Charalambous et al. (2014) [37] Cyprus	What are the attitudes and referral patterns of lung cancer specialists to palliative care?	50 cancer specialists from the EORTC Lung Cancer Group representing 14 countries.	Survey	Oncologists like to refer to palliative care when they have difficult to control symptoms or when no treatment is available. Some refer during the diagnosis of metastatic disease or during cancer treatment. Non-availability of palliative care physicians is a major barrier. Oncologists feel that patients do not like to be referred to palliative care and referral means abandoning their patients. Moreover, oncologists have concerns about expertise of palliative care physicians and feel that they may discourage or interfere with oncological treatment.	29
Cherny et al. (2003) [38] Israel	What is the practice and attitudes of ESMO oncologists in relation to the supportive and palliative management of patients with advanced and incurable cancer and what are the oncologist-related barriers to the provision of optimal supportive and palliative care?	895 ESMO member oncologists representing 64 countries.	Survey	All advanced cancer patients should receive concurrent palliative care. Oncologists should coordinate the care at all stages of disease including end of life care. Prefer to manage advanced cancer and dying patients themselves. They felt they have acquired some knowledge of palliative care during their training and feel confident about managing symptoms. Palliative care physicians do not have enough understanding of oncology to counsel patients with advanced cancer regarding treatment options.	33
Morikawa et al. (2016) [39] Japan	What are the barriers to collaboration between haematologists and palliative care teams in relapse or refractory leukaemia and malignant lymphoma patients’ care?	11 Haematologists from University Hospital	Content Analysis	Barriers for referral were a. treatment provided by palliative care providers were not as preferred by the haematologists b. treatment of primary disease given priority over palliative care c. Lack of aggressive approach by the palliative care team d. negative image of palliative care, palliative care is equated by the patients to imminent death e. palliative care team may not be able to support the haematology patients and they have a different perspective on assessment f. lack of interdisciplinary communication and g. lack of palliative care human resources.	25
Johnson et al. (2008) [40] Australia	What are the triggers that initiate referral to palliative care and what are the reasons for non-referral?	699 cancer specialists practicing at various hospitals in Australia	Survey	Patients with advanced cancer should be referred early. Early referral is beneficial, and patients will benefit from palliative care while receiving anti-cancer treatment. Patients are often referred when they have a terminal illness, uncontrolled physical symptoms, complex patient needs and when they are not coping with the physical care. They prefer integrated care and would like to be involved in the care even after palliative care referral. They are confident about managing symptoms and unlikely to refer if patient’s symptoms are well controlled and prognosis is good.	34
Ward et al. (2009) [41] Australia	What are the attitudes of Australian medical oncologists towards palliative care and collaboration with palliative care services?	115 Medical Oncologists who are members of Medical Oncology Group of Australia.	Mixed Method (Survey and thematic analysis)	Provision of palliative care is central, rewarding and adds value. Have adequate palliative care training and confident in managing symptoms and communication. Palliative care rotation should be part of medical oncology and vice-versa. Major reason for referral is symptom management, community support, hospice and terminal care. Prefer concurrent model over sequential model. Barriers to referral are, a. inadequate palliative care resources b. refusal by palliative care to take patients receiving anti-cancer therapy c. lack of clear guidelines regarding timing of referral	33
Hay et al. (2017) [42] USA	What are the Gynaec-oncologist’s views that influences the utilisation of outpatient specialist palliative care?	34 Gynaec-oncologists working at NCI designated cancer centres	Grounded theory	Long term relationship with the patients helps in convincing the patients to access palliative care. They value the communication skills of the palliative care provider, emotional support, help in navigating difficult circumstances, goals of care discussion and prognostic awareness. Prefer embedded clinics and better inter-disciplinary communication. Have concerns about losing control and awareness of patients receiving palliative care.	27
Wright et al. (2017) [43] UK	What are the views and perceptions of haematologists towards palliative care and the factors that helped or hindered referral to palliative care?	8 Haematologists working in a tertiary referral cancer centre	Grounded theory	Equated palliative care with worsening prognosis, death and dying. Hospice has a negative connotation, and hospice referral requires sensitive and proactive explanation. Symptom control was the most common reason to refer. Barriers for referral were a. inadequate number of palliative care beds b. palliative care lack resources to provide blood products c. inflexible referral criteria d. prejudice by the palliative care providers against haematology patients d. difficult timing and transition due to complex and unpredictable nature of illness e. uncomfortable to refer curative patients f. mixed messages g. loss of control g. sense of professional failure h. feelings of abandoning patients. Prefer joint care approach.	32
Smith et al. (2012) [44] USA	What influences lung cancer physician’s decision to refer their patients to experts in palliative care?	155 Lung Cancer Physicians practising at various teaching hospitals in USA	Survey	Patients and families would be alarmed by mention of palliative care. Patients prefer to focus on curative therapies than palliative care. Patients do not want to discuss prognosis. Palliative care providers are good in discussing complex issues and goals of care. They help in reducing symptoms, provide spiritual support and decrease the length of stay.	32
Rhondali et al. (2013) [45] USA	What are medical oncologists’ perceptions of the supportive care service and whether changing the name “palliative care” to “supportive care” influenced communication with patients and their families about palliative care and the referral?	17 medical oncologists working in a tertiary referral cancer centre	Grounded Theory	Symptom control is the primary function of palliative care. Palliative care communication helps in transitioning patients to end of life care. Psychological support provided by palliative care decreases caregiver anxiety and facilitates communication. Palliative care involvement has time saving benefit and helps patients to complete treatment due to better symptom control. Earlier referral is better as it will facilitate better therapeutic relationship. Triggers for referral are terminal nature of illness, metastatic disease and exhaustion of treatment. Barriers for referral are no clear-cut point to stop treatment, communication involved in referral is challenging, conflicts in goals of care and physician ownership. Prefer name to be changed as supportive care as patients perceive it better and will be more receptive for referral.	33
Schenker et al. (2014) [46] USA	What are the oncologist factors that influence referral decisions?	74 Oncologists practising at various hospitals in USA	Qualitative data analysis	Palliative care is an alternative to chemotherapy and cannot have both. Palliative care referral decision based on disease stage and treatment option. Palliative care has a different philosophy of care not compatible with active disease modifying care. PC providers create conflict and provide dismal prognosis. Referral to palliative care means abandoning, giving up. They are territorial and would like to provide treatment till the end and do not like others interfering with the care. They have palliative care skills and refer patients for symptom control. Having more A positive referral experience may facilitate referral.	25
LeBlanc et al. (2015) [47] USA	What are the differences in referral practice and views of palliative care among haematologists and solid tumour oncologists?	23 Haematologists 43 Solid tumour oncologists practicing at academic cancer centres	Mixed Method Study (Survey + Qualitative Data Analysis)	Haematologists view palliative care as end of life care and as antithetical to cancer care. Solid tumour oncologists feel palliative care expertise is useful and believe in co-management of patients. Haematologists expressed distrust, need to maintain control, avoiding involvement of other consultants, mixed messages, and prognostic uncertainty. Palliative care is inconsistent with treatment goals and a major barrier for referral. Solid tumour oncologists felt lack of palliative care resources, logistics of accessing, insurance as barriers for referral	28
Wentlandt et al. (2012) [48] Canada	What are the referral practices of Canadian oncologists to palliative care, particularly with respect to the timing of referral and to identify factors that were associated with timely versus late referral?	603 medical, radiation and surgical oncologists from the Canadian oncology societies	Survey	Diagnosis of an incurable cancer, uncontrolled physical symptoms and prognosis less than 1 year were the likely reason for referral. Referrals are also made for discharge planning and psychosocial support. The term palliative care has a negative perception and would refer if the name is changed to supportive care. They will refer the patients early if there are more palliative care services and palliative care providers accept patients receiving chemotherapy.	32
Wentlandt et al. (2014) [49] Canada	What are the attitudes and referral practices of paediatric oncologists to specialized palliative care services and to compare their practices and opinions with those of adult oncologists?	48 Paediatric Oncologists & 595 Adult Oncologists practising at various hospitals in Canada	Survey	Paediatric oncologists refer terminally ill patients, metastatic disease, prognosis of less than 6 months, and with uncontrolled symptoms to palliative care. Paediatric oncologists feel palliative care has a negative perception and prefer name change to supportive care. They are comfortable treating advanced patients at end of life. Adult oncologists refer earlier, when prognosis is < 1 year, for discharge planning, psychosocial and spiritual support.	30
Suwanabol et al. (2018) [50] USA	How surgeons who care for patients with colorectal cancers approach end-of-life care and engage palliative care specialists?	131 Cancer Surgeons belonging to the American Society of Colon & Rectal Surgeons	Mixed Method Study (Survey + Qualitative Data Analysis)	Oncologists have lack of knowledge, training and opportunities for delivery of palliative care. Barriers are inadequate communication between teams, unrealistic expectation in a poor prognosis situation and effectiveness of treatment, uncertainty in decision making, legal liability, opioid phobia, family conflict, lack of time, lack of palliative care services and culture of continuing life sustaining treatment in the hospital.	28
Gidwani et al. (2017) [51] USA	What are the oncologist’s views and attitudes towards palliative care referral?	31 Medical Oncologists practicing at academic cancer centres and Veteran Affairs hospitals in USA	Thematic Analysis	Palliative care is an important layer of support, should be provided early and unavailability of the palliative care provider is the barrier. Excessive focus on inpatient palliative care and absence of outpatient and community palliative care is the barrier. Palliative care provider is perceived as outsider, unable to understand oncology patients, oncology treatment and unable to recognise a recoverable sick oncology patient from a dying patient. Issues with care coordination and oncologists being left out of crucial discussions and lack of interdisciplinary communication is a barrier for referral. Palliative care has a narrow focus and palliative care providers excessively rely on sedation and analgesics.	25
Cripe et al. (2019) USA [52]	What are the perceived barriers to integrate specialty palliative care into gynaecologic cancer care?	174 Gynaec-oncologists who are members of the Society of Gynaecologic- Oncology in USA	Survey	Barriers to specialty palliative care in Gynaecologic Oncology practice were unrealistic expectations of families and patients, limited access to specialty palliative care, poor insurance reimbursement, time constraints, and concern of reducing hope or trust.	34
NyirÖ et al. (2018) [53] Hungary	What is the timing and circumstances of implementing paediatric palliative care in the Hungarian paediatric oncology practice?	22 physicians from the Hungarian Paediatric Oncology Group	Survey	Barriers for early palliative care implementation were increase in parental anxiety, detrimental effects on the doctor-family-patient relationship and equating palliation with end-of-life care. Oncologists preferred discussing with the parent’s first before referral, gradual information disclosure, and waiting till the end of child’s life for making referral. They preferred to have a psychologist in the earlier discussions. Avoided using the term palliative care and used euphemisms to communicate death/dying.	33
Ethier et al. (2018) [54] Canada	What are the various perceived barriers for having goals of care (GOC) discussions and palliative care referral?	127 oncologists from 12 cancer centres in Ontario, Canada	Survey	Barriers were patients have difficulty in accepting prognosis, and desire for aggressive treatment due to inflated expectation of treatment benefit. Other barriers were lack of time to have conversations, prognostic uncertainty, desire to maintain hope, uncertainty of the benefits of further active cancer therapy, difficulty in recommending discontinuation of treatment in younger population and patient and family refusals	34
Feld et al. (2019) [55] USA	What are the practices and attitudes regarding the role of early palliative care referral among oncologists and patients with metastatic non-small cell lung cancer?	279 Oncologists from the International Association for the Study of Lung Cancer	Survey	Common factors influencing participants to refer patients to palliative care were inadequately managed pain, no further or dwindling treatment options, other cancer-related symptoms, depression/anxiety. The common reasons for not referring patients to palliative care include lack of time to address palliative care needs lack of patient symptoms, belief that oncologists can manage palliative care needs independently, not wanting to burden patients with additional appointments, concern that referral may not be well-received by patients, and long wait times.	33
Prodhomme (2018) [56] France	What are the perceptions of haematologists on end of life discussion in patients and families with relapsed haematological malignancies?	10 haematologists from haematology centres of northern France and Belgium	Grounded Theory	Reluctance to discuss death and dying. Talking about death and dying is stressful, difficult and a taboo. Primary role is to cure and save from death. Responsibility is to reassure, motivate and infuse confidence. End of life discussion is not compatible with the physician’s role. Treatment effectiveness is proof of physician’s performance and death is a professional failure. End of life discussion causes loss of physician credibility and jeopardises patient compliance. Will initiate discussion only when there is an explicit request and would like to protect patients from violent discussions on end of life. Initiating palliative care and end of life care is incompatible with hope. Palliative care can be initiated when all the treatment is discontinued.	32
Tricou (2019) [57] France	What are the barriers for referring to specialist palliative care from the perspectives of the haematologists?	14 haematologists from two haematology centres at Lyon, France	Grounded Theory	Referral only when there are difficult to control symptoms like severe pain, complex situations and therapeutic decision making. Early referral beneficial in an asymptomatic patient for facilitating smooth transition. The term palliative care has a negative connotation and patients/families react negatively to palliative care. Preferred to use the term supportive care instead of palliative care. Short time frame from appearance of symptoms and end of life hinders referral	28
Sarradon-Eck (2019) [58] France	What are the perceptions and attitudes of oncologists on early referral to palliative care and working with the palliative care services?	13 Oncologists working at 10 cancer treatment sites across France	Grounded Theory	The term palliative care has a negative connotation. The term is unsuitable, scares people, harms them. Term palliative care elicits feelings of abandonment, stopping treatment and euthanasia. Prefer to use euphemisms life supportive care, comfort care instead of palliative care. More likely to refer if it is called supportive care. Referral to palliative care causes loss of patient compliance, loss of hope, elicits distress and equivalent to announcing poor prognosis. Palliative care is a last recourse and restricted for patients with less than three months life expectancy. Palliative care assessment is vague, not evidence based, and oncologists are experienced in symptom management.	28

### Review themes

Five themes developed during synthesis. They were a) presuppositions of oncologists and haematologists, b) power relationships and trust issues, c) making a palliative care referral: a daunting task, d) cost-benefit of a palliative care referral, and e) strategies to facilitate a palliative care referral. The themes, subthemes, and the explanatory narrative for the subthemes along with the citations are displayed in Table 3**,** and the thematic map of the review is visually represented in Additional file 5.

**Table 3:** Systematic Review Themes and Subthemes

Themes	Subthemes	Explanatory Narrative
1. Presuppositions of oncologists and haematologists about palliative care referral	Role conflict	Handing over patients to palliative care is a professional failure (Wright, 2017 [43]) Amounts to letting down and failing the patients (Wright, 2017 [43]) Treatment effectiveness is a proof of physician’s performance (Prodhomme, 2018 [56]) Primary role is to cure and save from death (Prodhomme, 2018 [56]) Medical training geared towards cure or control of the disease (Horlait, 2016 [36]) End of life discussion is not compatible with the oncologist’s role (Prodhomme, 2018 [56]) End of life discussion causes loss of physician credibility (Prodhomme, 2018 [56])
	Abandonment	Will be viewed by families as abandonment (Schenker, 2014 [46]) (Charalambous, 2014 [37]). Sense of abandonment when the focus of care was changed (Wright, 2017 [43]). Announcing poor prognosis signified abandonment (Sarradon-Eck, 2019 [58])
	Rupture of therapeutic alliance	Breakdown of doctor-patient relationship. (NyirÖ et, 2018 [53]) Jeopardises patient compliance (Sarradon-Eck, 2019 [58]) (Prodhomme, 2018 [56])
	Loss of hope	Desire to maintain hope is lost (Ethier, 2018 [54]) (Sarradon-Eck, 2019 [58]) Initiating palliative care is incompatible with hope. (Prodhomme, 2018 [56]) Responsibility to reassure, motivate and infuse confidence (Prodhomme, 2018 [56]) Protect patients from violent discussions on end of life (Prodhomme, 2018 [56])
	Negative emotions	Emotional toll while making palliative care referral (Wright, 2017 [43]) (Sarradon-Eck, 2019 [58]) Emotional burden associated with delivering news of poor prognosis (Sarradon-Eck, 2019 [58]) Emotional bond associated long term knowing of the patients (Horlait, 2016 [36]). Inability to handle emotional reactions associated with palliative care referral (Horlait, 2016 [36])
	Self-efficacy	Symptom management, psychosocial support and communication is integral part of oncology and can provide ourselves (Schenker, 2014 [46]) (LeBlanc, 2015 [47]) (Feld, 2019 [55]) (Sarradon-Eck, 2019 [58]) Oncologists have training in managing physical and psychological symptoms, and communicating with patients and families (Cherny, 2013 [38]) (Johnson, 2008 [40]) (Ward, 2009 [41])
2. Power relationships and trust issues	Control and coordinate the care process	Be responsible for the care of the patient till the end (Horlait, 2016 [36]) (Schenker, 2014 [46]) (Rhondali, 2013 [45]) (Hay, 2017 [42]) Coordinate the care of the patient at all stages of the illness (Cherny, 2013 [38]) (Wright, 2017 [43]) Referral leads to loss of control (Hay, 2017 [42]) (LeBlanc, 2015 [47]) Dislikes interference in patient care (Schenker, 2014 [46]) (Rhondali, 2013 [45]).
	Gatekeeping	Wait till the end of potential curative treatment to make referral (NyirÖ et, 2018 [53]) Referral only when all the treatment is discontinued (Prodhomme, 2018 [56]) (Sarradon-Eck, 2019 [58]) Initiate palliative care discussion only when there is an explicit request (Prodhomme, 2018 [56])
	Competency based trust	Treatment provided by palliative care not as expected (Morikawa, 2016 [39]) Palliative care providers not skilled in managing side effects (Morikawa, 2016 [39]) Unable to differentiate a recoverable sick patient from a dying patient (Gidwani, 2017 [51]) Do not have adequate oncology knowledge to counsel patients (Cherny 2003 [38]) Lack aggressive approach (Morikawa, 2016 [39]), (Schenker, 2014 [46]). Excessive focus on pain medications and sedation (Gidwani, 2017 [51]) Palliative care providers have a different perspective on assessment (Morikawa, 2016 [39]) Palliative care assessment is vague and not evidence based (Sarradon-Eck, 2019 [58])
3. Making a palliative care referral: A daunting task	Stigma associated with palliative care	Palliative care referral equals death, end of life, terminal care (Morikawa, 2016 [39]) (NyirÖ et, 2018 [53]) (LeBlanc, 2015 [47]) (Horlait, 2016 [36]) (Cripe, 2019 [52]) Negative perception about palliative care precludes the use of the term (Horlait, 2016 [36]) (NyirÖ et, 2018 [53]) (Tricou, 2019 [57]) (Sarradon-Eck, 2019 [58]) (Wentlandt, 2012 [48]) (Wentlandt, 2014 [49]) The term palliative care is unsuitable, scares people, harms them. (Sarradon-Eck, 2019 [58]) Elicits feelings of abandonment, stopping treatment and euthanasia (Sarradon-Eck, 2019 [58]) Use supportive care and comfort care instead (NyirÖ et, 2018 [53]) (Sarradon-Eck, 2019 [58]) Reluctance to discuss death and dying (Prodhomme, 2018 [56]) Discussing death is stressful and a taboo (Prodhomme, 2018 [56]) (Horlait, 2016 [36]) (NyirÖ et, 2018 [53]) Patients and families get alarmed by mention of palliative care (Smith, 2012 [44]) Physicians have to address the negative connotation associated with palliative care (Hay, 2017 [42]) Palliative care referral requires careful explanation (Wright, 2017 [43]) Referring to palliative care means patients are weak and unable to fight the disease (Rhondali, 2013 [45])
	Illness and treatment related factors	Advanced, recurrent or metastatic cancers are referred (Rhondali, 2013 [45]) Unpredictable course of illness hinders referral (Morikawa, 2016 [39]) Relapsing and remitting nature of haematological malignancies is a barrier for referral (Wright, 2017 [43]) Rapid progression and complications hinder referral (Wright, 2017 [43]) Short time frame from onset of symptoms to end of life is a constraint for referral (Tricou, 2019 [57]) Prognostic uncertainty is a barrier (Ethier, 2018 [54]) Referred when prognosis is poor (Wentlandt, 2012 [48]), (Wentlandt, 2014 [49]) Uncertainty in decision making hinders referral (Suwanabol, 2018 [50]) No referral if general condition is good (Horlait, 2016 [36]). Uncontrolled symptoms facilitate referral (Rhondali, 2013 [45]), (Wentlandt, 2012 [48]), (Wentlandt, 2014 [49]) (Feld, 2019 [55]) No referral in the absence of symptoms (Johnson, 2008 [40]), (Wentlandt, 2014 [48]) (Feld, 2019 [55]) No referral until treatment failure or late stage of the disease (LeBlanc, 2015 [47]). No referral if possibility of cure exists (Johnson, 2008 [40]) (Wright, 2017 [43]) (Ethier, 2018 [54]) Lack of treatment options is a trigger for referral (Feld, 2019 [55]) Priority on treatment of the disease and cure until the end hinders referral (Morikawa, 2016 [39]) Difficulty in recommending discontinuation of treatment in younger population (Ethier, 2018 [54])
	Patient and family attitudes	Unrealistic expectation of cure and desire for aggressive treatment (Suwanabol, 2018 [50]) (Horlait, 2016 [36]) (Ethier, 2018 [54]) (Cripe, 2019 [52]) Unwilling to discuss prognosis and non-curative approach (Smith, 2012 [44]) (Ethier, 2018 [54]) (Johnson, 2008 [40]) Unwilling to discuss referral to palliative care (Horlait, 2016 [36]) (Ward, 2009 [41]) (Charalambous, 2014 [37]) (Feld, 2019 [55]) Family conflict (Suwanabol, 2018 [50]) Cultural barriers (Suwanabol, 2018 [50]) (Horlait, 2016 [36]) Language barriers (Horlait, 2016 [36]) Negative public perception about death (Suwanabol, 2018 [50])
	Organisational Challenges	Hospital culture directed towards cure (Suwanabol, 2018 [50]), (Horlait, 2016 [36]) Lack of time to discuss about palliative care (Suwanabol, 2018 [50]), (Horlait, 2016 [36]) (Cripe, 2019 [52]) (Ethier, 2018 [54]) Lack of space to discuss about palliative care (Horlait, 2016 [36]) Documentation deficiencies in oncology case notes (Horlait, 2016 [36]) Legal liability of palliative care referral (Suwanabol, 2018 [50]) Fear of opioids and its side effects (Suwanabol, 2018 [50]) Lack of knowledge among the oncologist’s about palliative care (Suwanabol, 2018 [50])
	Lack of referral criteria	Not sure when to refer (Morikawa, 2016 [39]) (Wentlandt, 2012 [48]) (Charalambous, 2014 [37]) Lack of consensus among the oncologists about when to refer (Horlait, 2016 [36]) Lack of clear guidelines for palliative care referral (Ward, 2009 [41]) (Rhondali, 2013 [45]) (Wright, 2017 [43])
	Limited palliative care resources	Inadequate access to palliative care services (Johnson, 2008 [40]), (Suwanabol, 2018 [50]) (Cripe, 2019 [52]) Inadequate number of palliative care providers (Charalambous, 2014 [37]), (Morikawa, 2016 [39]). Inadequate palliative care resources (Wright, 2017 [43]), (Ward, 2009 [41]), (Gidwani, 2017 [51]) Long waiting times for palliative care appointments (LeBlanc, 2015 [47]) (Feld, 2019 [55]) Palliative care with excessive inpatient focus, lack of outpatient clinics (LeBlanc, 2015 [47])
4. Cost-benefit of palliative care referral	Benefits of referral	Pain and symptom control (Wright, 2017 [43]), (Schenker, 2014 [46]), (Rhondali, 2013 [45]), (LeBlanc, 2015 [47]), (Charalambous, 2014 [37]), (Smith, 2012 [44]) (Feld, 2019 [55]) (Tricou, 2019 [57]) Psychosocial support, managing depression and anxiety (Rhondali, 2013 [45]), (Smith, 2012 [44]) (Johnson, 2008 [40]) (Hay, 2017 [42]) (Feld, 2019 [55]) Improvement in quality of life (Gidwani, 2017 [51]), (Johnson, 2008 [40]) Navigating complex situations and therapeutic decision making (Tricou, 2019 [57]) (Hay, 2017 [42]) Prognostication and Goals of care discussion (Hay, 2017 [42]), (Smith, 2012 [44]) Conflict resolution (Hay, 2017 [42]) Saves oncologist’s time (Rhondali, 2013 [45]) Facilitating treatment completion by better symptom control (Rhondali, 2013 [45]) Survival benefit (Rhondali, 2013 [45]) Discharge planning (Wentlandt, 2012 [48]) (Wentlandt, 2014 [49]) Reducing the length of hospital stay (Smith, 2012 [44]) Community support (Ward, 2009 [41])
	Incongruent communication	Confusing and overwhelming communication (Wright, 2017 [43]) (Morikawa, 2016 [39]) (Schenker, 2014 [46]), (LeBlanc, 2015 [47]) Incongruent perception of prognosis by the palliative care providers. Information provided to the patients and families is not consistent with oncologist’s views (LeBlanc, 2015) [47], (Gidwani, 2017) [51] (Schenker, 2014 [46]) (Rhondali, 2013 [45]) Not including oncologists in goals of care discussion and advance care planning (Gidwani, 2017 [51])
	Curtailment of care	Reluctance of palliative care to consult haemato-oncology patients (Wright, 2017 [43]) Patients unable to access blood products while receiving palliative care (Morikawa, 2016 [39]) Reluctance of palliative care to consult patients receiving active anti-cancer treatment (Ward, 2009 [41]), (Suwanabol, 2018 [50]) Cost and burden of additional consultation (Feld, 2019 [55])
5. Strategies to facilitate palliative care referral	Integrated model of care	Concurrent anti-cancer treatment and palliative care (Cherny, 2003 [38]) (Wentlandt, 2012 [48]) (Ward, 2009 [41]) Co-management of patients (LeBlanc, 2015 [47]) (Johnson, 2008 [40]) (Ward, 2009 [41]) Complementary role (Gidwani, 2017 [51]) Embedded into oncology clinics (Hay, 2017 [42]) Palliative care rotation should be part of oncology training and vice-versa (Charalambous, 2014 [37]) (LeBlanc, 2015 [47]) Communication between the oncology and palliative care teams (Hay, 2017 [42]), (Suwanabol, 2018 [50]), (Ward, 2009 [41]) Clarification and assignment of roles and responsibilities (Ward, 2009 [41]) Rapport building and smooth transition (Schenker, 2014 [46]) (Tricou, 2019 [57]) (Rhondali, 2013 [45]) (Wentlandt, 2014 [48]), (Ward, 2009 [41]) (Johnson, 2008 [40])
	Changing the name	Changing the name to supportive care (Rhondali, 2013 [45]) (Tricou, 2019 [57]) (Sarradon-Eck, 2019 [58]) Referral likely if the name is changed to supportive care (Wentlandt, 2012 [48]) (Wentlandt, 2014 [49]) (Sarradon-Eck, 2019 [58])
	Resource augmentation	Availability of services (Schenker, 2014 [46], (Cherny, 2003 [38]) Consistency and continuity of care (Ward, 2009 [41]) Presence of in-patient palliative care beds (Ward, 2009 [41])

### Presuppositions of oncologists and haematologists about palliative care referral

Data from this review suggests that oncologists and haematologists perceived palliative care referral as role conflict, abandonment, rupture of therapeutic alliance and loss of hope. Making a palliative care referral triggered negative emotions, and they felt that they have the self-efficacy to manage palliative care needs. These perceptions informed some of the tendencies that might have hindered palliative care referral.

Some studies reported views on role conflict [36, 43, 56]. Haematologists felt that their role was to cure and save their patients, and end of life discussions was not compatible with their role [56]. Haematologists viewed palliative care referral as a therapeutic failure and letting down the patient and their families [43]. Moreover, haematologists also felt that engaging with palliative care reflected poorly on their performance and credibility [56].

Oncologists and haematologists expressed views on abandonment [37, 43, 46, 58]. British haematologists and the French oncologists felt that their patients and families experienced abandonment when there was a change in focus of care and during discussing poor prognosis [43, 58].

Some studies reported views on the rupture of the therapeutic alliance [53, 56, 58]. Hungarian paediatric oncologists felt that parental anxiety associated with early palliative care referral impeded the therapeutic relationship [53]. Some French oncologists and haematologists felt that palliative care referral could jeopardise both the doctor-patient relationship and compromise treatment compliance [56, 58].

Oncologists and haematologists equated palliative care referral to loss of hope [54, 56, 58]. A desire to maintain hope was a barrier for palliative care referral among the Canadian and French oncologists [54, 58]. The French haematologists felt the need to reassure and inspire confidence in their patients and protect them from any information that caused a loss of hope [56].

Making a palliative care referral could trigger negative emotional reactions among some oncologists and haematologists [36, 43, 58]. French oncologists felt emotionally burdened while delivering the news of poor prognosis [58]. Belgian oncologists and British haematologists felt that close emotional bond resulted from knowing their patients over time [36, 43]. It made them emotional while making a referral. Moreover, some Belgian oncologists found it challenging to handle the emotional reactions of patients and families associated with palliative care referral [36].

Seven studies supported the view that oncologists and haematologists perceived they have the self-efficacy to manage palliative care needs [38, 40, 41, 46, 47, 55, 58]. Oncologists and haematologists felt that managing symptoms, providing psychosocial support and communicating with the patients are integral aspects of oncology and they felt that they had developed these skills in their oncology training [38, 40, 41].

### Power relationships and trust issues

Data from this review suggests that oncologists and haematologists had the power to gatekeep the referral process and believed in competency-based trust. Many studies supported the view that oncologists and haematologists preferred to regulate the referral process and liked to control and coordinate the care process of their patients at all stages of their illness trajectories [36, 38, 42, 43, 45, 46]. The American oncologists perceived palliative care referral as loss of control [42, 47], and interference in the care process [45, 46]. In some studies, oncologists and haematologists liked to decide the timing of the referral [53, 56, 58]. Hungarian paediatric oncologists and the French oncologists and haematologists preferred to wait till the end of cancer treatment to make a palliative care referral [56, 58].

Synthesis of data from some studies shows that oncologists and haematologists have diminished trust in the competency of palliative care providers [38, 39, 46, 51, 58]. Japanese haematologists and French oncologists felt that palliative care providers have suboptimal assessment and management skills [39, 58]. Medical oncologists worldwide felt that the oncology training of palliative care providers was inadequate [38]. American oncologists questioned the reliability of palliative care providers as they were unable to differentiate a recoverable sick patient from a dying patient [51].

### Making a palliative care referral: a daunting task

Data from this review suggests that oncologists and haematologists perceive the task of making a palliative care referral as daunting. They had to deal with the stigma associated with palliative care, navigate illness and treatment associated factors, address patient and family attitudes, and overcome organisational challenges. Moreover, a lack of referral criteria and limited palliative care resources made the referral process even more challenging.

Most studies supported the view that oncologists and haematologists had to deal with the stigma associated with palliative care. Oncologists and haematologists had to deal with the public stigma of palliative care due to the negative stereotyped association of palliative care with death [36, 39, 47, 52, 53]. European oncologists and haematologists felt that families were reluctant to discuss death and viewed discussing death as a taboo [36, 56, 56]. Moreover, families felt stressed during the end of life discussions and equated these conversations with abandonment and euthanasia [56, 58].

Oncologists and haematologists also had to deal with label avoidance stigma or the choice not to pursue a line of management due to stigma associated with the language used in relation to the illness or treatment [36, 48, 49, 53, 57, 58]. French oncologists felt that the term palliative care has a potential to induce fear [58], and the American lung cancer specialists believed that patients and their families get alarmed on mentioning palliative care [44]. British haematologists and American gynae-oncologists felt that the term palliative care required careful explanation, and they had to dispel negative connotations associated with the term [42, 43]. Hungarian paediatric oncologists and French oncologists suggested changing the term to comfort care or supportive care [53, 58].

In most studies, oncologists and haematologists elucidated illness-related factors either facilitating or hindering palliative care referral. Stage of the illness [45], its course [39, 43, 57] and complications [43] determined palliative care referral. Prognostication [48, 49, 54], cure potential [39, 40, 43, 54], availability of treatment options [47, 55], and decision-making challenges [50] also played an important role in palliative care referral. Presence of symptoms [40, 45, 48, 49, 55] and performance status [36] of the patient were the other factors determining palliative care referral.

Data from this review suggest that oncologists and haematologists felt that beliefs and expectations of patients and families hindered palliative care referral. Unrealistic expectation of cure [36, 50, 52, 54], unwillingness to discuss prognosis and non-curative approach [40, 44, 54], and reluctance to discuss palliative care referral [37, 41, 55] were certain attitudes that hindered palliative care referral.

In this review, oncologists and haematologists had to overcome organisational challenges that hindered palliative care referral. A lack of space and time to discuss palliative care was a significant barrier [36, 50, 52, 54]. American surgical oncologists and Belgian oncologists felt that hospital culture directed towards cure hindered referral [36, 50]. American surgical oncologists feared legal liability of palliative care referral and expressed reservations about opioid use [50]. Both American medical and surgical oncologists believed that insufficient documentation about a patient’s illness in the medical records constrained palliative care referral [36, 50].

Lack of palliative care referral guidelines [41, 43, 45], and uncertainty about when to refer [37, 39, 48] constrained palliative care referral. Belgian oncologists felt that lack of consensus among the oncologists about the timing of referral was also a limiting factor [36].

In this review, oncologists and haematologists perceived that palliative care resources are limited. Inadequate palliative care resources [41, 43, 51], fewer palliative care providers [37, 39], and limited access to palliative care [40, 50, 52] hindered palliative care referral. American oncologists felt that long waiting times for palliative care appointments and limited palliative care outpatient clinics were some of the other barriers for referral [47, 55].

## Cost-benefit of palliative care referral

In this review, oncologists and haematologists have elucidated both the rewards and negative effects of making a palliative care referral. Pain and symptom management and psychosocial support are the well-known benefits of palliative care referral. In many studies included in this review, the oncologists and haematologists subscribe to the views on well-known benefits of palliative care referral. However, only a few studies elucidated lesser-known benefits of palliative care like facilitating goals of care discussion and shared decision-making. The Australian and American oncologists felt that palliative care improved the quality of life of their patients [40, 51]. Palliative care referral facilitated decision-making [42, 57] and enabled goals of care discussion [42, 44]. It also facilitated treatment completion [45], reduction in hospital stay [44], discharge planning [48, 49] and support for the patients in the community [41]. Oncologists and haematologists also experienced some direct benefits as it helped them to resolve family conflicts and saved the time of the oncologists [42, 45].

In this review, oncologists and haematologists felt that they had experienced some unintended negative outcomes of palliative care referral. In some studies, oncologists and haematologists felt that lack of congruence in communication between them and palliative care providers led to patients and families receiving mixed messages. They felt that palliative care communication could make patients and their families confused and overwhelmed [39, 43, 46, 47]. Moreover, they felt that palliative care providers have incorrect perception about the course of illness and treatment outcomes leading to inconsistencies in communication [45–47, 51]. The American oncologists emphasised the presence of an oncologist during the initial family meeting after palliative care referral [51].

Oncologists and haematologists felt that palliative care referral could risk curtailment of care of their patients. British and Japanese haematologists felt that palliative care providers are reluctant to consult haematology patients and the patients were unable to access blood products while receiving palliative care [39, 43]. The Australian medical oncologists and the American surgical oncologists felt that reluctance of palliative care providers to consult patients receiving active anti-cancer treatment could deprive management of these patients with palliative care needs [41, 50].

## Strategies to facilitate palliative care referral

In half of the studies included in the review, oncologists and haematologists provided some strategies that could facilitate palliative care referral. Oncologists and haematologists felt that strategies like developing an integrated model of care, changing the name of palliative care and augmenting palliative care resources could facilitate palliative care referral.

Oncologists and haematologists thought that integrated model of care could be achieved by providing concurrent cancer treatment and palliative care [38, 41, 48], co-management of the patients [40, 41, 47] and by having an excellent inter-team communication [41, 42, 50]. American oncologists felt that oncology and palliative care have complementary roles and preferred palliative care teams to be embedded in oncology clinics [42, 51]. Lung cancer specialists from Cyprus and American oncologists felt that palliative care rotation should be part of oncology training [37, 47]. In some studies, oncologists and haematologists felt that rapport building between the palliative care team and patient and their families is essential to facilitate smooth transitions of care from oncology to palliative care [40, 41, 45, 46, 49, 57]. Some oncologists and haematologists supported changing the name of palliative care [45, 57, 58]. They felt that palliative care referral is likely to improve if the term palliative care is replaced with supportive care [48, 49, 58]. Oncologists and haematologists felt the need to improve the availability of services, consistency and continuity of care and more in-patient palliative care beds [38, 41, 46].

### Subgroup analysis

Oncologists versus haematologists, adult versus paediatric physicians, and countries were the three subgroups that were analysed. Five studies included in this review captured the views of haematologists [39, 43, 47, 56, 57]. The haematologists had a higher negative perception of palliative care due to the term palliative care associated with death. They were less likely to refer and referred late due to complex unpredicted course of haematological illness and complications. Two studies captured the views of paediatric oncologists [49, 53]. Like haematologists, the paediatric oncologists also preferred to avoid the term palliative care. They also favoured waiting till the end of cancer treatment to make palliative care referral. The views of oncologists and haematologists across various countries included in the review were comparable.

## Discussion

In a cancer care setting, oncologists and haematologists have the discretionary authority to make treatment decisions for their patients and act as gatekeepers [59]. Their views impact palliative care referral. In this review, few oncologists and haematologists expressed their concern regarding referee’s lack of professional competency and referrer’s self-efficacy to meet various palliative care needs. Competency-based trust is an expectation that the other person can perform a task effectively, and reduced trustworthiness can hinder engagement with a person or a service [60]. The discourse of oncologists and haematologists in this review in terms of power to control and coordinate the care of their patients and determining the timing of referral suggests power dependence in a relationship. Power relationships exist when a person is dependent on another for the things that they value [61]. Palliative care is an end speciality, and its providers are dependent on oncologists and haematologists for a referral [62]. Moreover, the public stigma and label avoidance stigma due to negative stereotyped association of palliative care with death gave them the stigma power, where the stigmatisers had the power to exclude the stigmatised [63].

In this review, oncologists and haematologists have drawn symbolic inferences about palliative care referral. They felt that palliative care referral symbolised the loss of hope, abandonment, break in the therapeutic relationship and role conflict. Symbolic perspectives could influence human cognition and motivation and might act as internal reinforcement affecting the referral behaviour [64].

An integrated approach is to bring together and align professional inputs, services and clinical management [65]. In this review, few oncologists have advocated for an integrated model of cancer care where cancer care and palliative care is provided concurrently. Moreover, they felt that oncologists and palliative care providers have a complementary role where oncologists provide cancer treatment and palliative care providers manage symptoms and improve quality of life. The integrated approach is not limited to making a referral or transfer of patient information [66]. It involves providing critical inputs that could change the attitudes of health care providers [67]. In this review, changing the name of palliative care and rebranding it as supportive care is a crucial input provided by the oncologists, which might enable integration. Moreover, palliative care rotation being part of oncology training and palliative care providers having knowledge and skills in oncology were the other inputs that could facilitate integration. Palliative care participation in the multidisciplinary cancer meetings and coordination of care between the two teams can assist integration [68–70]. Moreover, having a standardised care pathway contextualised to the region or country might facilitate integration [71]. In this review, oncologists emphasised the need for excellent inter-team communication and palliative care embedded in the oncology clinics as a means to achieve integration.

### Social exchange theory as a framework for interpreting review findings

The process of referral is a social action where there is a temporary or permanent sharing of responsibility for patient care between the referrer and the referee [72]. Making a referral is a social behaviour [72]. Social Exchange Theory (SET) is the theorisation of social behaviour [73]. It is viewed in terms of social actors interacting to meet their needs, and the purpose of the interaction is to seek reward and avoid the cost [23]. The initial SET proposed by Homans was limited to the task, rewards and cost of interaction [74]. However, it was expanded by Blau and Emerson to include power relationships and cognitive perceptions of the social actors [61, 75]. The referral behaviour is usually based on referrer’s presuppositions towards the referee, the power relationship between the referrer and referee, the task of referral, rewards accrued, costs incurred and equity of relationship [76]. Presuppositions are emotions or feelings, which is an internal response to a social event [64]. Sentiments are affective states of emotion that have an evaluative role and influence social action [77, 78]. Therefore, presuppositions about palliative care referral formed tendencies, which had the potential to influence referral behaviour [24, 79].

According to SET, power and status of actors are considered as key factors determining the nature of exchange [77]. In this review, a few oncologists have requested the palliative care providers to change their name [45, 48, 49]. This request to rebrand [80] may also suggest a form of power imbalance as few services have conceded to that request [81]. Homans focused on the power of reward in social exchange [74]. In this review, the reward of palliative care was restricted to a couple of roles like pain management and psychosocial support. A study that used SET to understand non-terminal palliative care referral practices for Parkinson’s disease patients showed that endorsement of the rewards of palliative care referral by the neurologists was one of the strong predictors of referral [82]. The focus of Emerson’s modification of SET moved beyond the power of reward into coercive power [61]. Coercive power is the ability to control the negative events or the costs of exchange by the social actors in power advantaged position [61]. Inconsistencies in communication, curtailment of care and the challenges associated with accessing palliative care were perceived negatively by the oncologists that hindered future referral.

The review findings pertaining to the stigma of palliative care referral and stigma as a barrier to social exchange were the other perceptions explored using the SET. Cook and Emerson’s work on SET added cognitive perspectives and social structures as important components of exchange [83]. It also facilitated elucidating the individual and organisational factors in this review hindering palliative care referral. According to Emerson, in an exchange relationship, the power inequalities between the social actors can be balanced by coalition formation, division of labour and network extension [61]. Coalition formation is a mechanism by which a social actor in a less powerful position can gain advantage through collaboration [61]. In this review, few oncologists have advocated for an integrated model of cancer care where cancer care and palliative care is provided concurrently. The network extension is where the less powerful actor balance power by adding new partners to facilitate exchange [61]. The division of labour is where each social actor works according to his skills and specialisation towards fruition [61]. In this review, oncologists felt that co-management of patients by both the disciplines facilitated integration.

In this review, oncologists and haematologists offered some solutions to make this relationship equitable. Equity is appraising the rewards and costs on the background of sentiments, the intricacy of the tasks and outcomes [74]. According to SET, in an inequitable situation, people explore alternate choices, compare the present situations with alternate choices and may leave the situation [23]. However, in this review, oncologists offered strategies to facilitate palliative care referral instead of leaving the relationship. Sometimes people remain in the relationship even when they are inequitable for reasons that go beyond simple economic logic [84]. For oncologists and haematologists not referring to palliative care may not be an option, as there are limited alternatives to palliative care for a patient with advanced cancer. Therefore, “finding equitable solutions” can be added to the social exchange theory alongside “comparison level for alternatives” [23].

### Limitations and strengths

Three survey studies had a low response rate [37, 38, 40]. It could create a potential response bias as the respondents who completed this survey were the ones who are familiar with palliative care. Although they were low response surveys, each of these studies had a significant number of participants who provided views on facilitators and barriers for specialist palliative care referral. Three studies had respiratory physicians, colorectal surgeons and internists as participants along with oncologists [37, 40, 44]. Although some of the participants in these studies were not oncologists by training their views were included in the review as they were actively involved in the cancer care of the patients. Moreover, it was difficult to disaggregate their role in these studies. A few studies exploring the views of cancer providers about palliative care referral were excluded [9, 25, 85–91], as these studies had a heterogeneous mixture of physicians, physician assistants, nurses and social workers. It would not have been possible to disaggregate the physician views from the other healthcare provider’s views. The year of publication of the studies included in the review ranged from 2003 to 2019. However, most of the included studies were published in the last five years. The review findings of the earlier studies may not truly represent the contemporary attitudes of oncologists and haematologists towards palliative care referral.

The presence of two reviewers enabled comparison of the search results, identifying methodological strengths and weaknesses, and it facilitated the synthesis of the findings in a transparent manner. Disagreements were resolved by mutual consultation. The search terms were finalised after consulting with an information assistant. The articles included for the review were selected after appraisal, checking for relevance and scoring for methodological rigour. The synthesis was conducted systematically according to the narrative synthesis steps. Despite a few limitations, the themes derived from the systematic review were able to answer the review question satisfactorily. Findings from the surveys, qualitative studies and mixed methods studies mirrored each other, adding to the strength of the synthesis. The review had a mixed typology of studies, and the participants had diverse oncology backgrounds, traversing four continents. This heterogeneity added depth to the findings and their generalisability.

## Conclusion

The findings of this review suggest that some oncologists and haematologists liked to control and coordinate the care of their patients at all stages of illness trajectories and determine the timing of referral. They considered palliative care referral as abandonment, a break in the therapeutic relationship and loss of hope. They also expressed concerns regarding the professional competency of the palliative care providers and felt that they had the self-efficacy to manage the palliative care needs. Although illness-related factors acted as triggers for palliative care referral, the stigma associated with palliative care, patient and family attitudes, organisational challenges, lack of referral guidelines and limited palliative care resources made referral a daunting task. The findings of this review suggest that the majority of oncologists appreciated the pain and symptom management and psychosocial support role of palliative care. Lesser-known roles of palliative care were seldom elucidated. Some oncologists and haematologists felt that palliative care referral comes with a cost due to incongruencies in communication and curtailment of care. They felt that an integrated model of care, changing the name of palliative care and augmenting palliative care resources might facilitate a referral.

### Future considerations

In this review, views of paediatric oncologists are underrepresented as only two studies included in this review had their views. Therefore, there is scope for future research to know the views of paediatric oncologists and haematologists on palliative care referral. Moreover, a review exploring the views of patients and family caregivers on palliative care referral, and views of palliative care providers on receiving a referral from oncologists and haematologists may further bridge the knowledge gaps in palliative care referral.

### Implications for policy and practice

Review findings have several implications on policy and practice. There is a need for palliative care trainees to have training in oncology, and likewise, there is a need for oncology trainees to have palliative care training. Palliative care providers regularly joining the multidisciplinary team meetings might provide an excellent opportunity for both the teams to bond and build confidence, which could better inter-team communication. Moreover, to facilitate integration, a rebranding strategy is probably required.

## Supplementary information

Additional file 1. Search Terms.

Additional file 2. List of Journals Hand Searched.

Additional file 3. Hawker’s tool used for assessing the methodological rigour of the included studies in the review.

Additional file 4. Data Extraction Form.

Additional file 5. Thematic Map of Review Findings.

## Data Availability

All data generated or analysed during this study are included in this published article [and its supplementary information files].

## Abbreviations

CINAHL: Cumulative Index of Nursing and Allied Health Literature
EMBASE: Excerpta Medica dataBASE
MEDLINE: Medical Literature Analysis and Retrieval System Online
PICo: Population, Phenomenon of Interest and Context
PRISMA: Preferred Reporting Items for Systematic Reviews and Meta-Analyses
PROSPERO: International Prospective Register of Systematic Reviews
SET: Social Exchange Theory
USA: United States of America
