# Supplementary material for: How views of oncologists and haematologists impacts palliative care referral: a systematic review
Source: BMC Palliat Care. 2020 Nov 23;19:175. doi: 10.1186/s12904-020-00671-5 (PMC7686696; doi:10.1186/s12904-020-00671-5)
Supplement: Supplementary file 1 — Search Terms. [file 12904_2020_671_MOESM1_ESM.docx]

# Supplementary File 1: Search Terms

| **Concepts** | **PubMed** | **CINAHL** | **PsycINFO** | **EMBASE** | **Free Text Terms** |
| --- | --- | --- | --- | --- | --- |
|  | MeSH terms | MH terms | DE terms | Emtree terms |  |
| **Concept 1**  Views | 1. Attitude of health personnel  2. Motivation  3. Emotions | 1. Attitude of health personnel  2. Perception  3. Health behaviour  4. Health beliefs | 1. Health personnel attitudes  2. Motivation  3. Health behaviour | 1. Health personnel attitude  2. Health behaviour  3. Health belief  4. Motivation | 1. Attitude*  2. Motivation*  3. View*  4. Perception*  5. Belief*  6. Behavio*  7. Perspective*  8. Opinion*  9. Think* Thought*  10. Feel*  11. Emotion* |
| **Concept 2**  Oncologists  Haematologists | 1. Neoplasms  2. Hematologic Neoplasms  3. Oncology Service, Hospital  4. Hematology | 1. Neoplasms  2. Hematologic Neoplasms  3. Oncologists  3. Oncology  4. Oncologic Nursing  5. Oncology care units | 1. Neoplasms  2. Oncology | 1. Neoplasm  2. Hematologic disease  3. Oncology  4. Oncologist  5. Cancer centre  6. Hematologist | 1. Cancer*  2. Oncologist*  3. Hematologist* OR Haematologist  4. Tumour* OR Tumor*  5. Malignan*  6. Oncology |
| **Concept 3**  Referral | 1. Referral and consultation  2. Practice patterns, physicians | 1. Referral and consultation  2. Practice patterns | 1. Professional referral  2. Professional consultation | 1. Patient referral  2. Professional practice  3. Integrated health care system | 1. Refer* Cordinat*  2. Consult*  3. Collaborat*  4. Integrat*  5. Simultaneous  6. Concurrent  7. “Shared care”  8. Combin*  9. Engage*  10. Cooperat*  11. Continu* |
| **Concept 4**  Palliative Care | 1. Palliative Care  2. Palliative Medicine  3. Hospice Care  4. Terminal Care  5. Home care services | 1. Palliative Care  2. Hospice Care  3. Terminal Care  4. Hospice and Palliative Nursing  5. Home health care | 1. Palliative Care  2. Hospice  3. Home Care | 1. Palliative therapy  2. Terminal Care  3. Hospice  4. Home care | 1. Palliat*  2. Hospice*  3. “Terminal Care”  4. “End of life care”  5. “Supportive Care”  6. “Supportive Oncology”  7. “Home care” |
