# Supplementary material for: How views of oncologists and haematologists impacts palliative care referral: a systematic review
Source: BMC Palliat Care. 2020 Nov 23;19:175. doi: 10.1186/s12904-020-00671-5 (PMC7686696; doi:10.1186/s12904-020-00671-5)
Supplement: Supplementary file 2 — List of Journals Hand Searched. [file 12904_2020_671_MOESM2_ESM.docx]

# Supplementary File 2: List of Journals Hand Searched

1. American Journal of Hospice and Palliative Medicine
2. Journal of Clinical Oncology
3. Oncologist
4. Annals of Oncology
5. Palliative and Supportive care
6. Supportive care in cancer
7. Journal of Pain and Symptom Management
8. BMC Palliative Care
9. Palliative Medicine
10. Journal of Palliative Medicine
11. Indian Journal of Palliative Care.
