# Supplementary material for: How views of oncologists and haematologists impacts palliative care referral: a systematic review
Source: BMC Palliat Care. 2020 Nov 23;19:175. doi: 10.1186/s12904-020-00671-5 (PMC7686696; doi:10.1186/s12904-020-00671-5)

# Supplementary File 3: Hawker’s tool used for assessing the methodological rigour of the included studies in the review


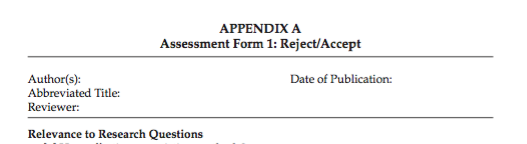


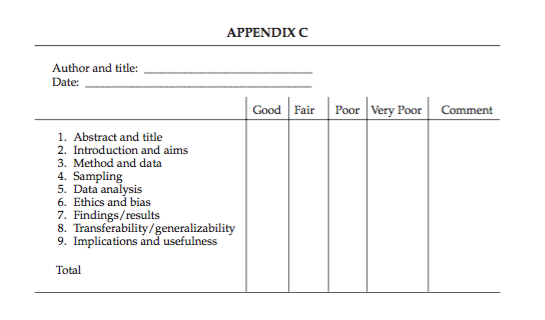


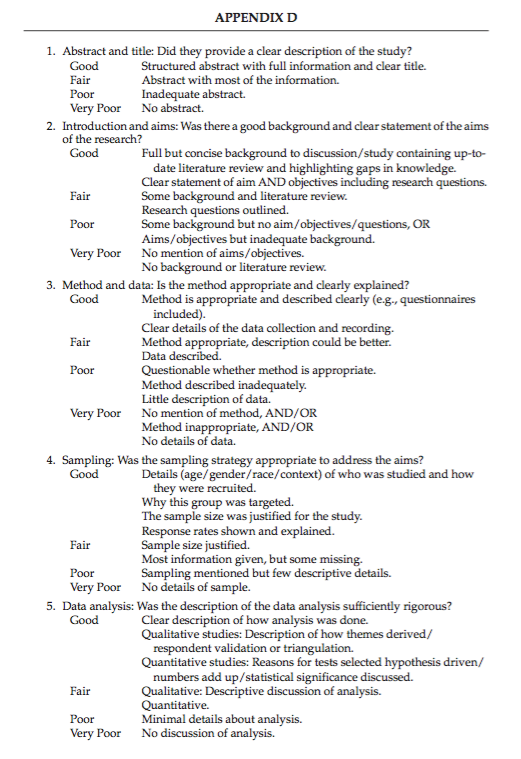


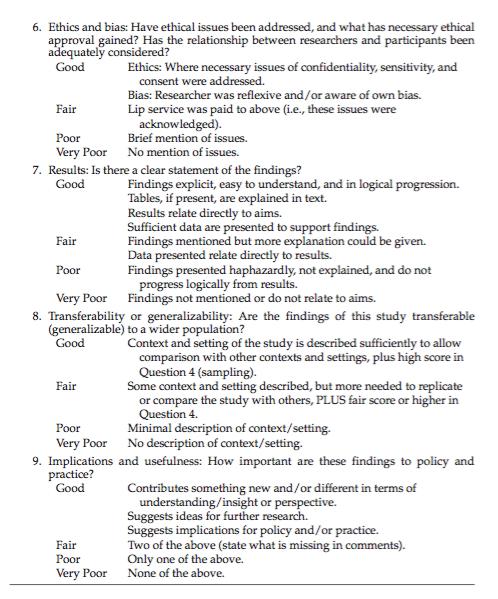

Supplement: Supplementary file 3 — Hawker’s tool used for assessing the methodological rigour of the included studies in the review. [file 12904_2020_671_MOESM3_ESM.docx]
