# Supplementary material for: How views of oncologists and haematologists impacts palliative care referral: a systematic review
Source: BMC Palliat Care. 2020 Nov 23;19:175. doi: 10.1186/s12904-020-00671-5 (PMC7686696; doi:10.1186/s12904-020-00671-5)
Supplement: Supplementary file 4 — Data Extraction Form. [file 12904_2020_671_MOESM4_ESM.docx]

# Supplementary File 4: Data Extraction Form

| **Section 1 General Information** | |
| --- | --- |
| Citation: | |
| Country: | Year: |
| Name of Reviewer 1: | Name of Reviewer 2: |

| **Section 2 Study Description and Population** | | | |
| --- | --- | --- | --- |
| Typology of evidence | Survey | Qualitative Study | Mixed Method Study |
| Research Question |  | | |
| Aim and Objectives |  | | |
| Study setting/context |  | | |
| Study duration |  | | |
| Target population |  | | |
| Role of the researcher in the study |  | | |

| **Section 3 Methodology** | |
| --- | --- |
| Sample size and sampling strategy  Appropriateness of sampling method and sample size |  |
| Participant recruitment |  |
| Characteristics of participants |  |
| Inclusion and Exclusion Criteria |  |
| Research Design |  |
| Theoretical underpinnings |  |
| Data Source |  |
| Methods/Tools used for data collection |  |
| Methods used for data analysis |  |
| Time points for measurement |  |
| Ethics approval |  |

| **Section 4 Findings** | |
| --- | --- |
| Outcomes measured |  |
| Key findings/Themes identified |  |
| Missing data |  |
| Reflexivity |  |
| Author’s conclusions |  |
| Author’s recommendation |  |

| **Section 5 Critical Appraisal** | |
| --- | --- |
| Strengths and Limitations of the study |  |
| Potential biases |  |
| Reviewer’s critical appraisal |  |
| Quality of the study  Reliability / Validity /Generalisability  Dependability / Credibility/ Transferability |  |
| Reviewer’s score |  |
| Agreement with another reviewer |  |
