# Supplementary figures and images for: How views of oncologists and haematologists impacts palliative care referral: a systematic review
Source: BMC Palliat Care. 2020 Nov 23;19:175. doi: 10.1186/s12904-020-00671-5 (PMC7686696; doi:10.1186/s12904-020-00671-5)

THEMATIC MAP OF  
REVIEW FINDINGS

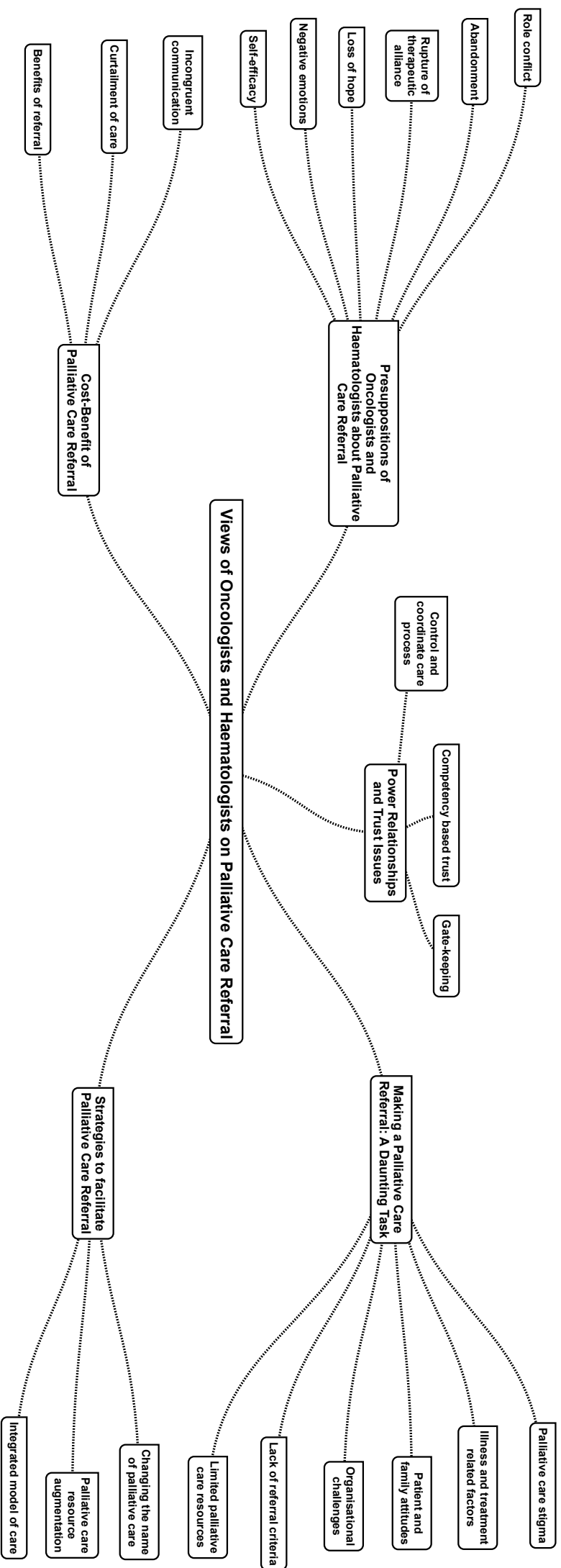

Supplement: Supplementary file 5 — Thematic Map of Review Findings. [file 12904_2020_671_MOESM5_ESM.pdf]
